# Relative and absolute inequalities in cerebrovascular disease mortality rates: exploring the influence of socioeconomic status and urbanization levels in Taiwan

**DOI:** 10.1186/s12889-024-18679-4

**Published:** 2024-04-27

**Authors:** Wen-Yu Lin, Ping-Yi Lin, Wen-Miin Liang, Hsien-Wen Kuo

**Affiliations:** 1 Institute of Environmental and Occupational Health Sciences, National Yang-Ming Chao Tung University, No.155, Sec.2, Linong Street, 112 Taipei, Taiwan; 2 Resource Circulation Administration, Ministry of Environment, Taipei, Taiwan; 3 Department of Nursing, Hung Kuang University, Taichung, Taiwan; 4 Department of Medical Research, China Medical University Hospital, Taichung, Taiwan; 5 Department of Health Services Administration, China Medical University, Taichung, Taiwan; 6 Institute of Public Health, National Defense University, Taipei, Taiwan

**Keywords:** Relative and absolute inequalities, Cerebrovascular disease mortality rates, Socioeconomic status, Urbanization

## Abstract

**Background/Objective:**

Limited evidence exists regarding the socioeconomic inequalities in cerebrovascular disease (CBD) mortality at different urbanization levels. Therefore, this study was conducted to assess the socioeconomic inequalities and urbanization levels in township-based CBD mortality in Taiwan.

**Methods:**

Socioeconomic variables, including the percentages of low-income households, individuals with a university education and above, and tax payments, were measured at the township level from 2011 to 2020. Urbanization was also determined by the national survey and divided into seven levels. Age-standardized mortality rate (ASMR) of CBD was calculated using a Geographic Information System (GIS) in 358 townships. The effects of socioeconomic variables and urbanization levels on relative and absolute inequalities in township-based CBD mortality rates were examined.

**Results:**

Significant differences in ASMR of CBD were observed across all socioeconomic status indicators over the years. Higher proportions of low-income households were associated with higher ASMR of CBD. Conversely, there were negative correlations between higher proportions of individuals with a university education and above and tax payments with ASMR of CBD. The regression analysis indicated significant impacts of relative and absolute socioeconomic inequalities on ASMR of CBD. Additionally, a moderation effect of socioeconomic variables and urbanization on CBD mortality rates was observed, with rural areas showing sensitivity to these factors.

**Conclusion:**

Although ASMR of CBD showed significant decreases over time, socioeconomic inequalities in CBD mortality rates persist. Interventions targeting socioeconomic inequalities in health outcomes, especially in rural areas, are needed to address this issue.

## Introduction

Cerebrovascular diseases (CBD), including stroke and other conditions affecting the blood vessels supplying the brain, are a significant public health concern worldwide [1]. The estimated global cost of stroke exceeds US$721 billion, which accounts for approximately 0.66% of the global GDP (Gross domestic product). Between 1990 and 2019, there was a significant increase in the burden of stroke in terms of the absolute number of cases, including a 70.0% rise in incident strokes, a 43.0% increase in stroke-related deaths, a 102.0% growth in prevalent strokes, and a 143.0% rise in Disability-Adjusted Life Years (DALYs). Notably, the majority of the global stroke burden, comprising 86.0% of deaths and 89.0% of DALYs, is concentrated in lower-income and lower-middle-income countries [2]. In Taiwan, CBD has been identified as one of the leading causes of death and is widely recognized as the primary cause of complex disability. The age-adjusted incidence of all strokes per 100,000 person-years decreased by 16%, from 251 (95% confidence interval [CI] 249–253) in 2004 to 210 (95% CI 209–212) in 2011 [3]. The burden of stroke in China is notably higher in comparison to certain high-income and developed countries like Europe and the United States [4]. This elevated burden contributes to a significant impact on disability-adjusted life years, indicating the substantial effect of stroke on the Chinese population. In 2019, the age-standardized incidence rate (ASIR) of stroke in China was 200 (with a confidence interval of 176–230), and the age-standardized mortality rate (ASMR) was 127 (with a confidence interval of 110–144). Over the period from 1990 to 2019, the ASIR for ischemic stroke had risen by 35.0% (with a range of 29.0–40.0%), while the ASMR had experienced a more modest increase of 3.0% (with a range from − 26.0 to 16.0%) [5]. The burden of CBD is not evenly distributed across populations, with certain socioeconomic and environmental factors playing a crucial role in determining disease outcomes [6]. Understanding the relationship between socioeconomic status (SES), urbanization, and ASMR of CBD is essential for developing effective public health strategies. SES indicators, such as income, education, and taxation, provide insights into the social and economic disparities that influence health outcomes [7]. Urbanization, on the other hand, reflects the level of development, infrastructure, and access to healthcare services within a region. A nonlinear single-threshold effect exists in the relationship between urbanization and population health indicators [8]. In a national health insurance cohort study that included 37,044 patients spanning from 2002 to 2013, the results revealed that patients residing in more advantaged regions within the middle-income category exhibited lower hazard ratios (HRs) for overall mortality. The 12-month hazard ratio (HR) was 1.27 (with a 95% CI of 1.13–1.44), and the 36-month HR was 1.25 (with a 95% CI of 1.14–1.37) when compared to those in disadvantaged regions with a 12-month HR of 1.36 (95% CI, 1.19–1.56) and a 36-month HR of 1.30 (95% CI, 1.17–1.44) [9].

Health inequality refers to the disparities in health outcomes and access to healthcare services among different population groups within a given society [10]. These inequalities can be influenced by various factors, including socioeconomic status (SES), education, occupation, and geographical location [7]. In Taiwan, like many other countries, health inequality is a significant public health concern. Despite the country’s overall high level of healthcare and strong healthcare system, certain population groups experience disparities in health outcomes and access to healthcare services. SES has been identified as a major determinant of health inequality in Taiwan, with individuals from lower socioeconomic backgrounds facing higher risks of poor health outcomes [11]. Taiwanese with lower SES have been found to have higher rates of chronic conditions such as cardiovascular diseases (CVD), diabetes, and lung cancer [12, 13]. Additionally, vulnerable populations, including indigenous peoples and rural communities, often face barriers to accessing healthcare services and experience higher rates of certain health conditions [3].

Understanding health inequality is essential for developing equitable healthcare policies and interventions to reduce disparities and improve Taiwanese health outcomes [3]. However, limitations in previous studies on health inequality in Taiwan include the cross-sectional design, small sample sizes, reliance on self-reported measures, limited focus on specific population groups, and insufficient consideration of contextual factors. By analyzing comprehensive data on ASMR of CBD, income levels, education attainment, taxation, and urbanization levels across different townships, we can gain insights into the complex interplay between these factors and CBD outcomes. Findings from this study will provide valuable information for policymakers, healthcare professionals, and researchers to develop targeted interventions aimed at reducing CBD burden and addressing health disparities within populations. Therefore, this study aims to explore the relative and absolute inequalities in ASMR of CBD concerning socioeconomic status and urbanization levels.

## Materials and methods

### Measurement of outcome

The ASMR data for 358 townships in Taiwan province spanning from 2011 to 2020 were obtained from the Center for GIS, RCHSS, Academia Sinica, and their Taiwan SMR Map. It’s worth noting that this dataset excludes 10 townships from Kin-men County and Lian-Jiang County due to the absence of urbanization-level information. The database of ASMR data is freely available on the Taiwan Mortality Rate Map website of the Center for GIS, RCHSS, Academia Sinica (http://me.geohealth.tw/). The population data used in the ASMR of CBD calculations is obtained from the population statistics of various counties and townships in the Social and Economic Database of the Ministry of the Interior. The standard population used in the calculations is the WHO 2000 World Population Standard. The ASMR of CBD is calculated using a method called direct standardization. The ASMR of CBD was calculated by the crude mortality rate for each age group in the township area. Calculate the crude mortality rate for each age group by dividing the number of deaths in that age group by the population and multiplying the crude mortality rate for each age group by the age-specific population ratio of the 2000 world standard population. This yields the ASMR of CBD for each year, gender, and township area level. By utilizing these calculations and data sources, the study obtains the ASMR of CBD, providing insights into the mortality patterns within that specific township.

### Definition of socioeconomic variables and levels of urbanization

While we have leveraged data from multiple township sources within the government, it is important to note that our protocol did not receive approval from the Institute Review Board (IRB). In the study, it was determined that there is a total of 358 townships in Taiwan Province, excluding the ten townships located on two outer islands. The proportion of low-income households in each township area was obtained from the Ministry of Health and Welfare’s Statistics Department. Based on the Social Assistance Law in Taiwan was officially implemented in 1998, the minimum living standard is defined as 60% of the average per capita consumption expenditure in the past year. Families with incomes below this minimum living standard are referred to as low-income households.

The database of low-income households data is freely available on the website of Ministry of Health and Welfare (https://www.mohw.gov.tw/dl-27852-9984ad06-c621-446e-bbf6-12e1de3ac350.html). The data provided by this department includes information on the number of low-income households in each township. To calculate the proportion of low-income households, the person number of low-income households in each area was divided by the total population of that township. Population data for each township area was sourced from the Ministry of the Interior’s Department of Household Registration. To calculate the proportion of the population with a university degree or above, data were obtained from the Government Open Data Platform (https://data.gov.tw/dataset/8409). This proportion is defined as the number of individuals in the educational attainment of population aged 15 and above with higher education, divided by the total population aged 15 and above in each township. The median tax payment data was derived from the statistical analysis tables of counties, cities, townships, and villages in the Statistical Monograph published by the Fiscal Information Agency, Ministry of Finance. The actual tax payment will be computed after taxpayers fill in the Alien Individual Income Tax Return Form for tax declaration. The database of median tax payment data is freely available on the website of Ministry of Finance (https://www.fia.gov.tw/WEB/fia/ias/isa109/ISA109.html). The median tax payment for each township or city area was calculated, and the unit used is in thousands of New Taiwan Dollars (NTD). Three socioeconomic variables of township areas in this study were categorized into three groups based on the proportions obtained for each variable: “low (≤ 25%)”, “medium (26%-75%)”, and “high (≥ 76%)”.

The levels of urbanization used in this study is derived from the “Taiwan Social Change Survey“(REF). This survey was initiated by the Ministry of Science and Technology, National Science Council in 1983. The survey follows the principle of conducting surveys every five years. In 2001, it joined the International Social Survey Program (ISSP) and began conducting annual surveys in Taiwan, synchronized with other member countries, starting in 2002. Additionally, since 2006, the survey has participated in the East Asia Social Survey (EASS), which includes Taiwan, South Korea, Japan, and China. The EASS conducts a joint thematic survey every two years. The survey employs strict quality control and comparison measures for interviewer surveys. It classifies the data based on six categories, which include the percentage of the commercial population, industrial population, population aged 15–64, population aged 65 and above, population with tertiary education or higher, and population density. To measure the differences within the same variable, the survey utilizes Ward’s Minimum Variance Method, with the squared Euclidean distance as the measurement distance method. Based on the clustering method, the survey classifies the 358 townships in Taiwan into seven clusters, including metropolitan core, industrial and commercial areas, emerging towns, traditional industry towns, low-developed townships, aging townships, and remote townships. These clusters provide a framework for understanding the degree of urbanization and the characteristics of different townships in Taiwan based on various socioeconomic indicators. Based on the seven levels of urbanization, we broadly classify areas into two categories: urban and rural.

### Statistical method

The data analysis was conducted using SPSS package version 24. Correlational analysis was utilized to describe the variations in socioeconomic variables and ASMR of CBD across four consecutive times (2011, 2014, 2017 and 2020). One-way ANOVA was employed to assess the ASMR of CBD among three levels of socioeconomic variables and seven levels of urbanization levels. To explore socioeconomic inequality in CBD mortality rates, the SII and RII were calculated. These indices were used to assess the statistical interaction effects between time and three socioeconomic variables on ASMR of CBD. Furthermore, the analysis examined the relationship between time, socioeconomic inequality, and ASMR of CBD based on urban and rural areas. The Slope Index of Inequality (SII) is determined through a regression analysis of ASMR of CBD related to socioeconomic variables known as ridit (relative to an identified distribution unit). Ridit values represent the midpoint of the range in the cumulative distribution of the population based on equity stratification. The SII quantifies the absolute disparity in ASMR of CBD between individuals with the lowest (bottom 10%) and highest (upper 10%) socioeconomic status. It is calculated as the absolute difference in ASMR of CBD. The magnitude of the SII is influenced by both the actual ASMR of CBD and the positioning of extreme socioeconomic groups, referred to as the “leverage” effect of values significantly distant from the mean. The Relative Index of Inequality (RII), developed by Kunst & Mackenbach [14] and Sergeant & Firth [15], is a ratio obtained from the regression-estimated mortality rates between socioeconomic groups with ridit values of 1 and 0. To calculate the RII, the regression line is extrapolated to theoretical extremes on the x-axis, representing values of 0 and 1. The RII is interpreted as a relative risk measure, incorporating information from ten-scale socioeconomic groups in assessing socioeconomic inequality. It is calculated by dividing the value at the bottom (10%) of the social hierarchy (intercept) by the value at the top (10%) of the hierarchy (intercept + slope). We computed both the RII and SII for each socioeconomic variable and also determined their corresponding confidence intervals (CI). Significance was established for distributions in which the 95% CI for the SII did not include 0 and the CI for the RII did not encompass 1.

## Results

Table 1 displays the annual trends in ASMR of CBD, the proportion of individuals with a university education or above, the proportion of low-income individuals, and tax payments across 358 townships in Taiwan across four consecutive years (2011, 2014, 2017 and 2020). The results indicate a consistent year-on-year decline in ASMR of CBD. In 2020, the ASMR of CBD was 15.5% lower than that of 2011. The proportion of low-income individuals in each township has also shown a decreasing trend over the years, with a 9.5% decrease in 2020 compared to 2011. The proportion of individuals with a university education or above has exhibited an increasing trend, with a 31.4% increase in 2020 compared to 2011. Regarding the historical trend in tax payments, there was an overall increase from 2011 to 2017. However, there has been a decrease in tax payments observed in 2020 compared to previous years.

**Table 1:** Trends in ASMR of CBD, the proportions of individuals with a university education or above and low income, and tax payments across 358 townships in Taiwan Province

	2011	2014	2017	2020	*P* trends
CBD mortality (1/100,000 people)	36.90 ± 16.07	36.64 ± 19.82	32.65 ± 16.87	31.19 ± 15.79 (-15.5%)*	< 0.001
Low-income (%)	2.10 ± 2.30	2.30 ± 2.30	2.00 ± 1.90	1.90 ± 1.70 (-9.5%)	< 0.001
Education (%)	16.9 ± 6.8	17.3 ± 7.8	19.8 ± 8.3	22.2 ± 8.5 (31.4%)	< 0.001
Tax payment (1,000 NT)	527.2 ± 70.0	565.0 ± 66.8	584.7 ± 64.7	407.1 ± 64.6 (-22.8%)	< 0.001

*Mean$\pm$SD (change % between 2011 and 2020)

Table 2 compares the trends in ASMR of CBD in three levels of socioeconomic indicators using a tertile approach. The results indicate significant differences in ASMR of CBD among all socioeconomic indicators over four years. Among the groups with higher proportions of low-income households, there is a tendency for higher ASMR of CBD. The group with the lowest proportion of low-income households had the highest decrease in ASMR of CBD in 2020 compared to 2011, with a decrease of 15.3%. Among the groups with higher proportions of individuals with a university education or above, there is a trend toward lower ASMR of CBD. However, the group with the highest proportion of individuals with a university education or above had a smaller decrease in ASMR of CBD in 2020 compared to 2011, with a decrease of only 6.9%, compared to 11.6% and 12.2% in the other two groups. Regarding the three groups based on tax payments, there is no clear trend. However, in both 2011 and 2020, higher tax payment groups had lower ASMR of CBD. The group with the highest tax payments had the highest decrease in ASMR of CBD in 2020 compared to 2011, with a decrease of 35.7%, while the decreases in the other two groups were 24.4% and 32.6%, respectively.

**Table 2:** Difference in ASMR of CBD (1/100,000 people) in three socioeconomic status indicators across four years

	2011	2014	2017	2020
Low-income (%)				
Low	31.71 ± 8.24	30.74 ± 7.59	28.12 ± 7.97	26.87 ± 9.35 (-15.3%)*
Medium	34.07 ± 12.07	33.07 ± 10.70	29.50 ± 9.08	27.34 ± 8.31(-6.73%)
High	49.26 ± 23.01	48.80 ± 32.27	43.59 ± 27.58	45.62 ± 24.76 (-7.4%)
Education (%)				
Low	45.52 ± 22.89	47.26 ± 29.75	46.35 ± 29.01	51.94 ± 26.75 (11.6%)
Medium	34.33 ± 8.71	32.57 ± 8.50	30.56 ± 9.70	30.15 ± 17.73(-12.2%)
High	27.21 ± 7.40	28.20 ± 7.27	26.58 ± 6.81	25.32 ± 6.40(-6.9%)
Tax payment				
Low	42.43 ± 17.90	30.61 ± 10.57	24.82 ± 27.60	32.08 ± 16.36(-24.4%)
Medium	33.39 ± 15.68	39.09 ± 21.08	34.38 ± 18.00	22.52 ± 6.08(-32.6%)
High	28.62 ± 8.06	32.17 ± 17.21	29.97 ± 14.42	19.11 ± 6.50(-35.7%)

*CBD mortality rates are significant differences in all variables (change % between 2011 and 2020)

Table 3 compares the trends in ASMR of CBD in seven levels of urbanization. The results indicate significant differences in CBD mortality rates among the seven urbanization levels during the four consecutive times. The ASMR of CBD are consistently lowest in the metropolitan core area, while the rates are higher in the outlying island townships. Across each urbanization level, there is a decreasing trend in ASMR of CBD over time. The remote townships exhibit higher variability in ASMR of CBD compared to other regions. In 2014, the ASMR of CBD in the remote townships was the highest at 73.75 per 100,000 people, which decreased to 57.90 per 100,000 people in 2020, representing a decrease of 21.5%. This decrease is higher than the 13.7% decrease observed in the metropolitan core area.

**Table 3:** The disparity in ASMR of CBD (1/100,000 people) across seven levels of urbanization

Urbanization levels	2011	2014	2017	2020
Metropolis Core(*N* = 25)	25.00 ± 6.82	24.95 ± 5.85	23.24 ± 5.06	21.54 ± 6.11 (-13.84%)*
Industrial and commercial urban(*N* = 40)	27.62 ± 5.67	28.27 ± 5.73	25.00 ± 5.24	23.69 ± 5.37 (-9.49%)
Emerging towns(*N* = 73)	35.47 ± 7.59	32.21 ± 6.92	30.02 ± 7.41	27.30 ± 5.73 (-15.40%)
Traditional industrial towns(*N* = 47)	36.08 ± 8.27	37.39 ± 11.60	33.26 ± 9.37	29.39 ± 10.68 (-7.82%)
Low-level development townships(*N* = 93)	36.02 ± 12.49	34.23 ± 12.10	31.70 ± 12.77	30.36 ± 11.54 (-12.00%)
Aging townships(*N* = 40)	37.00 ± 12.20	33.95 ± 11.04	30.07 ± 9.89	33.25 ± 10.69 (-18.72%)
Remote townships(*N* = 41)	63.24 ± 30.46	73.75 ± 40.48	59.23 ± 35.97	57.90 ± 31.00 (-6.34%)

*There are significant differences in all variables across seven levels of urbanization (change % between 2011 and 2020)

Based on Table 4, the study investigated the relationship between three socioeconomic indicators and ASMR of CBD, analyzing both the SII and RII. The socioeconomic status categories were divided into deciles. The results revealed that in townships with a higher proportion of low-income households, the ASMR of CBD tended to be higher. Specifically, the SII decreased from 2.334 per 100,000 people in 2011 to 2.097 per 100,000 in 2020, while the RII increased from 1.055 in 2011 to 1.057 in 2020. This suggests that the difference in ASMR related to CBD between townships with high and low proportions of low-income households has remained consistent over time. However, in the township with higher proportions of individuals with a university education or above, there is a trend toward lower ASMR of CBD. The SII decreased from − 2.495 per 100,000 people in 2011 to -2.866 per 100,000 people in 2020, and the RII decreased from 0.933 in 2011 to 0.919 in 2020, indicating a decreasing trend in the disparity between ASMR of CBD for education levels in townships with higher proportions of low-income households. Furthermore, in terms of tax payments, the SII decreased from − 2.035 per 100,000 people in 2011 to -2.031 per 100,000 people in 2020, while the RII decreased from 0.944 in 2011 to 0.926 in 2020. The RII showed a decreasing trend over time, indicating a growing disparity in ASMR of CBD related to the proportion of education and tax payment proportions. In summary, the findings demonstrate significant socioeconomic inequality in ASMR of CBD, and this inequality has been progressively worsening over the years.

**Table 4:** The absolute (SII) and relative (RII) inequalities in ASMR of CBD over time, using a ten-point scale for socioeconomic indicators

	2011	2014	2017	2020
Low-income (%)				
SII (95% CI)	2.334**(1.805—2.862)	1.981**(1.401—2.560)	2.217**(1.447—2.807)	2.097**(1.590—2.605)
RII (95% CI)	1.055**(1.044—1.067)	1.054**(1.040—1.069)	1.060**(1.045—1.074)	1.057**(1.044—1.081)
Education (%)				
SII (95% CI)	-2.495**(-3.045– -1.945)	-2.664**(-3.323—-2.005)	-2.327**(-2.888—-1.647)	-2.866**(-3.400—-2.331)
RII (95% CI)	0.933**(0.922—0.945)	0.933**(0.920—0.946)	0.935**(0.922—0.947)	0.919**(0.908—0.931)
Tax payment				
SII (95% CI)	-2.035**(-2.682— -1.389)	-1.003*(-1.908— -0.099)	-1.189**(-2.072 — -0.307)	-2.031**(-3.163 — -0.899)
RII (95% CI)	0.944**(0.930–0.958)	0.971*(0.951–0.991)	0.964**(0.943–0.986)	0.926**(0.899–0.954)

Note: *P < 0.05; **P < 0.01

Table 5 presents a regression analysis examining the relative (RII) and absolute (SII) inequalities in ASMR of CBD based on three socioeconomic variables and urbanization levels. First, for the proportion of low-income households, the highest urbanization level (metropolitan core) witnessed a 1.9% decline in RII and a decrease of 2.248 deaths per 100,000 people in ASMR of CBD compared to the lowest urbanization level (remote townships). Townships in the top 10% bracket for low-income households experienced a 4.8% increase in the ASMR of CBD in RII and a rise of 2.682 deaths per 100,000 people in SII compared to townships in the bottom 10% bracket for low-income households. Secondly, in terms of education level proportions, townships with remote townships had higher ASMR of CBD, with an increase in RII by 15.7% and an increase in SII by 7.438 deaths per 100,000 people. Compared to the lowest group, township with highest proportions (upper 10%) of individuals exhibited a decrease of 1.8% in RII and a decline of 2.192 deaths per 100,000 people in SII on ASMR of CBD. Lastly, regarding tax payments across different townships, remote townships had higher ASMR of CBD, with an increase in RII by 14.0% and an increase in SII by 5.518 deaths per 100,000 people. Highest tax payments (upper 10%) compared to the lowest group (bottom 10%) showed a decrease in SII by 0.828 deaths per 100,000 people. Significant statistical interactions were found between socioeconomic variables and urbanization on ASMR of CBD. The interaction effects between proportion of low-income households and tax payments with urbanization level were 0.934 and − 0.163 deaths per 100,000 people for the SII in ASMR of CBD, respectively. For townships with the highest proportion (upper 10%) of individuals with a university education or above and urbanization level (metropolitan core) compared to referent group, the RII stood at 0.985, while the SII was − 0.919 deaths per 100,000 population in ASMR of CBD. This suggests that there are significant variations in socioeconomic inequality in ASMR of CBD based on levels of urbanization.

**Table 5:** The relative (RII) and absolute (SII) inequalities of ASMR of CBD in relation to three SES indicators and urbanization levels

	Low-income^*^	Education^**^	Tax Payment^§^
Relative inequality			
Urbanization	1.157**(1.135 — 1.178)	0.981*(0.964— 0.998)	1.140**(1.122 — 1.160)
RII	1.018**(1.005 — 1.031)	0.952**(0.941 — 0.964)	1.006(0.996 — 0.959)
Urbanization X RII	0.985**(0.982 — 0.987)	1.019**(1.016— 1.021)	1.000(0.997 — 1.002)
Absolute inequality			
Urbanization	7.438**(6.543 — 8.334)	-2.248**(-3.040 —-1.455)	5.518**(4.470 — 6.295)
SII	2.192**(1.586 — 2.797)	-2.682**(-3.229 — -2.134)	-0.828**(-0.329 — -1.327)
Urbanization X SII	-0.919**(-1.039 — -0.799)	0.934**(0.818 —1.050)	-0.163**(-0.280 — -0.047)

*p < 0.05; **p < 0.01

^*^Township with the proportions of low-income households;^**^Township with the proportions of individuals with a university education or above; ^§^ Township with tax payment (1,000 NT)

When urbanization was categorized into two groups, urban and rural areas, Table 6 presents the relative (RII) and absolute (SII) inequalities in three socioeconomic variables in ASMR of CBD. Significant decreases in ASMR of CBD over time were observed. Townships with highest proportion (upper 10%) of low-income households was associated with significantly increased RII (1.018 for urban areas, 1.073 for rural areas) and SII in ASMR of CBD (0.566 per 100,000 people for urban areas, 3,306 deaths per 100,000 people for rural areas) compared to areas with a lowest proportion (bottom 10%) of low-income households. Conversely, education level and tax payment were negatively correlated with ASMR of CBD. Townships with the highest proportion (upper 10%) of individuals with a university education or above displayed significant impacts on lower ASMR of CBD: an RII of 0.943 for urban areas, 0.923 for rural areas, and an SII of -1.811 deaths per 100,000 people for urban areas, and − 3.552 deaths per 100,000 people for rural areas. Likewise, townships with the highest proportion of tax payment (upper 10%) exhibited significant impacts on lower ASMR of CBD, with an RII of 0.952 for urban areas, 0.956 for rural areas, and an SII of -1.521 deaths per 100,000 people for urban areas, and − 1,852 deaths per 100,000 people for rural areas. Overall, our findings indicate that socioeconomic inequality are associated with ASMR of CBD in Taiwan. Additionally, there seems to be a moderating effect of urbanization on ASMR of CBD. This effect is particularly noteworthy in townships with tax payment, where the SII is 0.179 deaths per 100,000 people, contrasting with urban areas where it is 0.092 deaths per 100,000 people.

**Table 6:** The relative (RII) and absolute (SII) inequalities of ASMR of CBD in relation to three socioeconomic indicators in both urban and rural areas

	Urban area			Rural area		
	Low-income^*^	Education^**^	Tax Payment^§^	Low-income^*^	Education^**^	Tax Payment^§^
Relative inequality						
Time	0.972** (0.964— 0.998)	0.994 (0.978 — 1.009)	0.958** (0.947 — 0.969)	0.978** (0.968— 0.990)	0.997 (0.985 — 1.009)	0.958** (0.946 — 0.970)
RII	1.018** (1.005 — 1.033)	0.943** (0.932 — 0.956)	0.952** (0.939 — 0.966)	1.073** (1.061 — 1.084)	0.923** (0.910 — 0.936)	0.956** (0.938 — 0.973)
Time X RII	1.002 (0.999— 1.004)	1.000 (0.998 — 1.002)	1.003** (1.001 — 1.005)	1.000 (0.999— 1.002)	1.000 (0.998 — 1.002)	1.004 (0.998 — 1.007)
Absolute inequality						
Time	-0.783** (-1.257 —-0.308)	-0.440 (-0.999 — 0.118)	-1.336** (-1.741 — -0.930)	-0.539 (-1.124 —0.047)	-0.320 (-0.938 — 0.298)	-1.693** (-2.293 — -1.092)
SII	0.566* (0.063— 1.069)	-1.811** (-2.261 — -1.360)	-1.521** (-2.011 — -1.030)	3.036** (2.470 — 3.603)	-3.552** (-4.274 — -2.830)	-1.852** (-2.701 — -1.003)
Time X SII	0.035 (-0.046 —0.116)	0.023 (-0.053 — 0.098)	0.092* (0.019 — 0.164)	-0.324 (-0.125 — 0.061)	0.061 (-0.052 — 0.173)	0.179** (0.056 —0.302)

*p < 0.05; **p < 0.01

^*^Township with the proportions of low-income households;^**^Township with the proportions of individuals with a university education or above; ^§^ Township with tax payment (1,000 NT)

## Discussion

In the early period, Hu et al. [16] reported that the decline in the age-adjusted death rate from CVD in Taiwan was not as rapid as in the United States and Japan, and the trends of CVD mortalities did not show steady decreases from 1972 to 1983. This decline can be attributed to improvements in healthcare, advancements in medical treatments, and better management of cardiovascular risk factors. However, our findings indicate a consistent year-on-year decline in ASMR of CBD from 2011 to 2020. In 2020, the CBD mortality rate was 15.5% lower than that of 2011. However, it is important to note that there may be variations within different townships or socioeconomic population subgroups within Taiwan. Our study aligns with the findings of Hu et al. [16], which indicated geographic differences in the downward trend of CVD death rates during that period. Evidence also suggests that the period and age trends of CVD rates differ between the working population and the overall Taiwanese population, with more severe relative effects observed in younger workers, particularly those aged < 55 years [17]. Compared to China, there has been a rapid increase in the ASIR of first-ever stroke in Taiwan. This increase is attributed to factors such as high fasting glucose levels, alcohol consumption, and obesity, particularly prevalent in low economic groups. Our findings indicate that socioeconomic inequality at the township level is associated with ASMR of CBD. Kivimäki et al. [18] demonstrated that women in low socioeconomic positions had a 2.3 times higher risk (95% CI 1.3–3.9) of CBD compared to those in high socioeconomic positions. Furthermore, after adjusting for conventional risk factors such as prevalent hypertension, coronary heart disease, diabetes, smoking, heavy alcohol consumption, physical inactivity, and obesity, the excess risk was attenuated by 23%. This suggests that socioeconomic disparities contribute significantly to the incidence and mortality of CBD. Similarly, Bray et al. [19] identified significant socioeconomic disparities in the burden of ischemic stroke and intracerebral hemorrhage in England. The disparities were particularly evident in stroke hospitalization risk and case fatality rates, and to a lesser extent, in the quality of health care. However, it is worth noting that this study lacked information on important stroke risk factors such as smoking status, body-mass index, or physical activity. Notably, the previous data used in these studies did not provide sufficient detail to assess the impact of socioeconomic disparities and urbanization on stroke risk. Further investigation is required to ascertain the precise mechanisms that link regional deprivation and medical factors to survival after the onset of ischemic stroke in low-income patients [9].

Our findings align with those of Manana et al. [20] conducted in Spain, which also demonstrated a negative association between CVD mortality and educational level. The study found that this inequality was more pronounced for premature mortality caused by cardiac conditions, such as ischemic heart disease and heart failure, particularly among women. The RII for all-cause CVD mortality was 1.88 (95% CI, 1.80–1.96) in women and 1.44 (95% CI, 1.39–1.49) in men. The SII was 178.46 deaths per 100,000 people for women and 149.43 deaths per 100,000 people for men. These results suggest that socioeconomic disparities play a significant role in CVD mortality rates, with higher CVD mortality rates observed among individuals with lower educational levels. It is worth noting that the niches of the study, we analyzed how the interaction between socioeconomic inequalities and ASMR of CVD evolves over time using panel data.

The CVDNOR project conducted in Norway [21], which investigated 141,332 incident cases of acute myocardial infarction (AMI) between 2001 and 2009, revealed significant relative differences in education-related inequalities, particularly among women aged 35–69. The incidence rate ratio (IRR) for individuals with basic education compared to those with tertiary education was 3.04 (95% CI 2.85–3.24), indicating a substantially higher risk among individuals with lower educational attainment. Additionally, the RII for education was 4.36 (95% CI: 4.03–4.71), demonstrating a strong social gradient in AMI incidence. However, it is worth noting that the relative inequalities did not show significant absolute changes from 2001 to 2009, suggesting that the disparities in AMI incidence based on education remained relatively stable over this time period [22]. Similarly, income inequalities in CVD also increased, with the RII for women rising from 1.52 in the 1990s to 2.62 in the late 2000s [23]. However, existing studies have not explored the interaction effects between socioeconomic variables and time periods on ASMR of CBD in urban and rural areas. Indeed, while the educational level is widely used to act as a comparable socioeconomic indicator, it is important to acknowledge that socioeconomic inequality encompasses various factors beyond education, including income, occupation, and other social determinants of health [24, 25]. Our study produced consistent results with prior research regarding the influence of socioeconomic inequality on CBD mortality. We observed significant associations between the proportions of low-income households, education levels with university or above, and tax payments with ASMR of CBD. Furthermore, it underscored the significance of the level of urbanization as a contributing factor to the overall prevalence of strokes in Taiwan [26]. Furthermore, we also identify interaction effects between socioeconomic inequality with urbanization and time, highlighting the complex interplay between these factors. These results align with previous studies [9, 13, 27] and underscore the importance of considering both socioeconomic factors and urbanization when examining CBD mortality in Taiwan.

Certainly, the evidence strongly supports the presence of an inverse social gradient and inequity gap, whereby mortality rates are highest among individuals in the poorest income group and decrease as income levels rise [28–30]. These trends align with the risk factors of CVD prevalence, particularly among individuals in lower-income groups residing in urban areas. These individuals tend to have a higher exposure to risk factors such as smoking, insufficient physical activity, and poor dietary behaviors, which in turn contribute to the development of obesity, diabetes, dyslipidemia, and hypertension [29, 30]. Indeed, considering the multifaceted nature of social determinants and their impact on CBD mortality is crucial for gaining a comprehensive understanding of health disparities. Each factor, including socioeconomic status, education level, income, occupation, access to healthcare, and lifestyles, can contribute to these disparities [26, 30]. To enhance our understanding of the complex interplay between socioeconomic factors and lifestyles with health outcomes, future research should prioritize the incorporation of longitudinal data. Longitudinal studies would allow for the examination of the temporal aspects of socioeconomic inequalities and their influence on CBD mortality over time. This would provide valuable insights into how social determinants may change or persist and their long-term impact on health outcomes. By examining the multifaceted nature of social determinants and their interaction with health outcomes, researchers can identify targeted interventions and policies to reduce health disparities and improve health equity. Moreover, it is crucial to examine the disparities between urban and rural areas, particularly the notion that rural populations are inherently healthier and live longer than urban populations. This examination should utilize current small geographical units with comparable size and population homogeneity, alongside the recently introduced Rural and Urban Area Classification, and various indicators of socio-economic variables [31]. The further research not only reaffirm the significance of different economic indicators and other causes of death including cancer, lung cancer, respiratory disease, circulatory disease, suicide, and accidents, but also delve into the relative and absolute economic disparities among different towns and villages on a national scale. These results can be replicated to corroborate previous studies [31]. Such research can inform public health strategies aimed at addressing the underlying socioeconomic factors contributing to CBD mortality and ultimately improving population health outcomes.

The niche of the study lies in investigating the socioeconomic inequalities in CBD mortality at different urbanization levels in Taiwanese populations. We aim to address the limited evidence on this topic and provide insights into the factors influencing ASMR of CBD. However, the study has some limitations. The study focuses on Taiwanese populations, and the findings may not be directly applicable to other regions or countries with different socioeconomic contexts and healthcare systems. The study relies on secondary data sources, such as socioeconomic variables measured at the township level and ASMR of CBD. The accuracy and completeness of these data may vary, which could potentially impact the study’s results and conclusions. Since the study utilizes aggregated data at the township level, it is susceptible to the ecological fallacy. Individual-level characteristics and behaviors are not directly examined, and conclusions are drawn based on associations observed at the group level. Therefore, individual-level relationships may not necessarily align with the findings of the study. The study primarily focuses on examining associations between socioeconomic variables, urbanization levels, and ASMR of CBD. While regression analysis is used to explore relationships, establishing causality is challenging due to the observational nature of the study. There may be other unmeasured factors or confounding variables that contribute to the observed associations. The study covers the period from 2011 to 2020, and the findings may not capture more recent changes or trends in ASMR of CBD and socioeconomic indicators. Long-term follow-up and analysis of more recent data could provide a more comprehensive understanding of the topic. For advancing research, Casant & Helbich [32] advocate for two main approaches. Firstly, they recommend conducting individual-level cohort and case-control studies across various sociocultural contexts. Secondly, they emphasize the complexity of both rurality and urbanicity, which cannot be adequately captured by oversimplified typologies. They suggest that detailed assessments of the sociophysical residential environment are necessary to fully understand these concepts. Therefore, further research with more diverse populations and robust study designs would be beneficial to enhance our understanding of socioeconomic inequalities in CBD mortality.

## Conclusions

Significant differences in ASMR of CBD were revealed across all socioeconomic variable and levels of urbanization. Higher proportions of low-income households in Taiwan’s township were associated with elevated ASMR of CBD, while higher proportions of education and tax payments were negatively correlated with CBD mortality rates. Regression analysis indicated RII and SII in ASMR of CBD based on socioeconomic variables and levels of urbanization. Particularly in rural areas, a moderation effect of socioeconomic variables and urbanization on ASMR of CBD was observed. These findings underscore the need for interventions targeting socioeconomic inequalities in health outcomes, with a particular focus on addressing this disparity in rural-urban areas.

## Data Availability

The datasets generated and/or analysed during the current study are available from open-access websites. The data can be assessed through the link, (http://me.geohealth.tw/) (https://www.mohw.gov.tw/dl-27852-9984ad06-c621-446e-bbf6-12e1de3ac350.html) (https://data.gov.tw/dataset/8409) (https://www.fia.gov.tw/WEB/fia/ias/isa109/ISA109.html).
